# Colonialism, malaria, and the decolonization of global health

**DOI:** 10.1371/journal.pgph.0000936

**Published:** 2022-09-06

**Authors:** Jesse B. Bump, Ifeyinwa Aniebo

**Affiliations:** 1 Department of Global Health, Harvard T.H. Chan School of Public Health, Boston, Massachusetts, United States of America; 2 Initiative on the Future of Health and Economic Resiliency in Africa, Boston, Massachusetts, United States of America; 3 Bergen Center for Ethics and Priority Setting, University of Bergen, Bergen, Norway; 4 Health Strategy and Delivery Foundation, Lagos, Nigeria; Royal Infirmary of Edinburgh and University of Edinburgh, UNITED KINGDOM

## Abstract

This paper explores the decolonization of global health through a focus on malaria and European colonialism in Africa. We employ an historical perspective to better articulate what “colonial” means and to specify in greater detail how colonial ideas, patterns, and practices remain an obstacle to progress in global health now. This paper presents a history of malaria, a defining aspect of the colonial project. Through detailed analysis of the past, we recount how malaria became a colonial problem, how malaria control rose to prominence as a colonial activity, and how interest in malaria was harnessed to create the first schools of tropical medicine and the academic specialization now known as global health. We discuss how these historical experiences shape malaria policy around the world today. The objective of this paper is to advance discussion about how malaria and other aspects of global health could be decolonized, and to suggest directions for future analysis that can lead to concrete steps for action.

## Introduction

What would it mean to decolonize global health? This simple question has become a primary feature of the field’s published discourse, drawing particular momentum as the COVID-19 pandemic has highlighted vast inequalities in the distribution of vulnerability, risk, and interventions such as vaccines [1–6]. More generally, awareness of inequalities within and between societies has led to questions about how to counter the sequelae of historic injustices, including slavery and colonialism. Motivation for these questions has included ongoing inequalities, as in the geography of power: most prominent among the donor countries are the former colonial and imperial powers, which also house leading institutions of research, education, philanthropy, commerce, and international governance. By contrast, formerly colonized countries remain poor, and formerly subjugated people enjoy worse health and shorter lives. Similarly, prominent journals and leading authors of global health research remain largely associated with the United States, the United Kingdom, and other colonial powers, even as their work is largely concerned with formerly colonized places and people. These and similar observations about the inequalities of influence and decision making have informed demand for the decolonization of global health [7–12].

The discussion of decolonization in global health has been conducted mainly with reference to unfair outcomes, and a specific agenda for decolonization has yet to be articulated. In part, this reflects the obviousness and simplicity of some problems, which do not require sophisticated analyses or call for complex solutions. For example, in April 2021, the US President’s Malaria Initiative announced a $30 million grant to seven institutions to help African governments improve data for decision making in malaria control and elimination. Yet none of the institutions were in Africa—they were in the US, the UK, and Australia. In voicing concerns about this, several African scholars working in malaria also noted that just 1% of research funding for malaria goes to African institutions; 99% goes to institutions based in rich countries where malaria is an academic specialty and not a public health problem [13]. However, the persistence of funding inequalities shows that unfairness alone is unlikely to change the processes that produce it. The imperative of decolonizing global health thus identifies the need to examine more closely what “colonial” means and to specify in greater detail how colonial ideas, patterns, and practices remain an obstacle to progress in the present.

## Historical methods

In this paper we seek to inform ongoing discussions of decolonization in global health by examining malaria and the history of its control. We selected malaria because it was a defining aspect of the colonial project and remains prominent in global health today. We choose to focus mainly on sub-Saharan Africa because that is where anti-malaria activities are most prominent now globally and where the burden of disease remains highest. This geographic emphasis is also justified by our interest in modern global health, which is most closely connected to the industrialized European colonization of Africa that began in the mid-19^th^ century. We recognize that this choice limits our ability to generate inferences about the consequences of other kinds of colonialism, such as settler colonialism. Establishing these boundaries allows us to address a series of questions that are otherwise too open-ended to answer clearly in a brief, exploratory paper. For example, what was and is colonial about malaria and its control? What connection is there between ideas and actions of the past and the ongoing present? How do these old ideas constrain our thinking now, and how can we make progress against malaria if colonial influences persist?

We present important aspects of the colonial history of malaria that continue to shape global health now. We explored the history of malaria and tropical medicine by reading journal articles from the late 19^th^ and early 20^th^ centuries, along with more recent scholarship by historians and other analysts of malaria in that period. Through detailed analysis of the past, this paper recounts how malaria became a colonial problem, how malaria control rose to prominence as a colonial activity, and how interest in malaria was harnessed to create the first schools of tropical medicine and the academic specialization now known as global health. We discuss how these historical experiences shape malaria policy around the world today and how the inequalities are perpetuated in the structure of global health. Our overall objective is to advance discussion about how malaria could be decolonized, and to suggest directions for future analysis that can lead to concrete steps for action.

## Colonization and decolonization

A major obstacle for advocates of decolonization in global health is the field’s very weak connections to history and the rich tradition of anti-colonial scholarship. It is not possible to decolonize without understanding what is colonial in the first place. We analyze the history of malaria to show how global health was developed to serve colonial interests and how it continues to embody colonial ideologies. For our purposes we define colonialism as the state-sponsored construction of non-merit inequality for the benefit of one group at the expense of another. As we show in this article, these non-merit inequalities circumscribe practically every aspect of global health, including in geography, knowledge, language, prominent journals, analytic methods, institutions, and categories of individual and group identity such as race, gender, ethnicity, and lived experience.

In opposing these non-merit inequalities we join a scholarly legacy as old as colonialism itself. Many before us have identified key features of coloniality and decolonization. Ngũgĩ wa Thiong’o has challenged the capacity of the English language to reflect African ideas and experiences [14]. Franz Fanon documented the centrality of racial discrimination to colonialism and theorized about the illegitimacy of European domination of other lands [15, 16]. Aníbal Quijano has questioned the Eurocentric core of ideas such as modernization [17]. Historians and other theorists in the Subaltern Studies collective have shown how to confront colonial narratives and assert agency for those colonizers sought to oppress [18]. Many scholars have analyzed the dependency created by colonialism and its detrimental implications for the present and future [19–21]. Similarly, political decolonization has not led to economic independence for African states, as Kwame Nkrumah argued in *Neocolonialism*: *The last stage of imperialism* [22]. The World Health Organization’s first African Regional Director for Africa Comlan A. A. Quenum warned that international institutions could not be allowed to become instruments of “recolonization” as he called for a “new economic order based on equity, equality, and the interdependence of all states” [23]. In this article we connect general themes of the decolonization literature with the specific example of malaria.

## Results and discussion

### An obstacle to colonization

The study of malaria and its control were so closely tied to colonization that these two legacies cannot be separated. The colonial project provided the reasons to study malaria, determined who was in a position to do so, and shaped knowledge generation and its application for malaria control, along with the distribution of its benefits [24–30]. As we show in detail below, the reason to study malaria was that it was the largest obstacle to colonization. Metropolitan military and business interests were compromised by the susceptibility of White settlers to malaria, which was by far the largest cause of death for that group. As historian Raymond Dumett has shown, in coastal cities such as Lagos and Freetown, White mortality averaged 70 or 80 per 1000 annually in the late 1800s, but colonizers in the interior fared much worse. In 1865 a British parliamentary committee had recommended largely withdrawing from West Africa altogether due to disease threats. When the Gold Coast was declared a colony in 1874, the first three candidates declined the Governor’s job because of the health risks; James Maxwell assumed the position on March 4th but died of malaria that same month [31]. Although reliable data are not widely available, some paint a devastating picture. For instance, for European troops in Sierra Leone from 1817–1838 average annual mortality was nearly 500 per 1000 [30].

The tremendous malaria mortality figures raise the question of motives for colonization. In the face of such fearsome odds of death, primarily from malaria, why did Europeans do it? The answer lies in perceived business opportunities and the development of state administrative mechanisms that favored some European interests, especially those in trade, shipping, and manufacturing using African materials. There had been substantial trade between Europe and Africa since at least the 1500s, centered mainly on slavery. When the international slave trade was banned by the British in the early 1800s it disrupted longstanding relationships that had been based mainly at European coastal forts. African rulers would sell captured enemies, among others, to European slavers at these places. As analyzed by economic historian Edward Reynolds, following the slave trading ban, Europeans sought trade in other products, including in raw materials such as cotton and gold, and in manufactured goods from Europe. Private trade favored an emerging class of African merchants who served as middlemen going between the coastal areas and customers living inland, where many Europeans would not venture due to the risks of malaria. Many African merchants had direct relationships with European manufacturers, but these and other arrangements were undercut by many disagreements [32].

As happened in other places, in the Gold Coast the British responded by trying to build legal and administrative structures that would let Europeans trade more easily and more directly with African customers, as Reynolds has analyzed. In economic terms, Chiefs had benefitted as the sellers of slaves, but trade in raw materials and products favored merchants, whose power grew over the early decades of the 1800s. Thus, at mid-century when the British proposed an alliance with Chiefs and pledged to establish a stable trade system, they found some agreement. To pay for the expenses of administration, the British asked Chiefs to submit to their rule and in 1852 announced a poll tax, non-payment of which was used to justify fines against Indigenous people. The right to collect the fines was then sold by the British government to private companies that wanted to be paid in local products such as palm oil. Ongoing disputes led to a series of Anglo-Ashanti wars and the declaration of a Gold Coast Colony by the British in 1874 [32]. Similar dynamics played out in other colonies, such as Nigeria [33], and as Elise Huillery has shown for French West Africa, colonial administrations were financed mainly via taxes on the colonized, rather than with metropolitan funds [34]. Thus, the main reason for colonization was to secure unfair trade advantages for European firms [32], although other motives such as religious conversion and racial discrimination were also prominent [35]. (There are many more comprehensive discussions of the political economy of colonization than is possible here [36–38]). The main obstacle to these objectives was malaria, which limited military control and threatened all European activities, especially in the interior. Hence, malaria became a critical threat to European colonial ambitions and a major priority for study and resolution.

### A colonial priority and an academic specialty

The academic field of global health is a direct descendant of tropical medicine, which was founded around the turn of the 20^th^ century by former military and colonial officers searching for pay and prestige beyond what their governments would offer. The initial story centered on malaria research, but even as the remit of global health has become much broader modern observers will recognize many patterns established at the foundation of tropical medicine. Among many others, this includes the location of leading research institutions in metropolitan cities, the close links between these schools and metropolitan governments, the weak accountabilities toward the places and people who were objects of study, an emphasis on research questions and metropolitan concerns rather than health more generally, and a complete ignoring of economic and political subjugation in favor of a focus on pathogens.

One starting point for the story of tropical medicine could be dated to 1880, when the causal agent of malaria (the plasmodium) was identified by a French Army physician working in Algeria, Alphonse Laveran [39, 40]. Mosquito transmission of malaria was demonstrated in the 1890s by Ronald Ross, a British colonial officer in the Indian Medical Service [41]. Both men felt their research contributions were undervalued by their respective colonial services, and both used their parasitological celebrity to transition to full-time research careers that were independent but still closely associated with metropolitan and colonial governments [42, 43].

These moves to create an independent specialty around the knowledge and experience of successful colonial medical offices reflected powerful forces that created the first schools of tropical medicine, still leading institutions in global health. The first two were founded in 1898 in Liverpool, which Ross joined the following year, and London. Liverpool had dominated the English slave trade, followed by London in second place [44, 45]. The Liverpool School of Tropical Medicine was founded with support from the Elder Dempster shipping company, revealing the critical importance of malaria control to the private sector businesses that profited from colonialism [29, 46]. In the late 1800s, “British trading firms and chambers of commerce [were] the leading critics of West African health conditions [and] harassed the Colonial Office with complaints about the polluted ponds and wells, refuse-strewn streets and yards, and open sewage pits” as major threats to their own health and the profitability of their businesses [31].

The London School of Tropical Medicine was founded with support from the Colonial Office and voluntary contributions from the British public. This reflected the combined interests of Medical Advisor to the Colonial Office Patrick Manson, who had proven the insect transmission of disease as a colonial officer in Southeast China in the 1870s and later mentored Ross, along with businessman and Secretary of State for the Colonies Joseph Chamberlain [28]. Chamberlain had presided over the launch of both schools and at Liverpool’s inauguration declared “The fight against tropical diseases constitutes the real basis of the politics of colonization” [29]. The government enfranchised the two schools by requiring that all newly recruited colonial medical officers receive training at either one [47], thus granting a duopoly on mandatory education and tying them closely to the colonial project.

This pattern was followed by all the major European colonial powers, which founded their own similar schools in the years that followed. As historian Isabel Amaral has analyzed in the case of a school of tropical medicine in Lisbon (founded 1902), medical authorities used similar arguments to gain support. In her retelling, one proponent in 1901 captured the sentiment as follows:

Colonisation is not only a social and economic question but also a question of hygiene and pathology. The prosperity and wealth of a colony depend, first of all, on the ease of the living conditions to be found there by the colonists. The remedy to the serious risks presented by colonization undertaken blindly lies in the intervention of medicine together with the highly powerful resources that are currently available. England, Germany and France have demonstrated their recognition of this reality by creating centres for study and teaching that can easily be converted into colonial well-being and colonial prosperity [25].

Malaria was the motive force behind the creation of academic tropical medicine, a blend of laboratory science, medicine, hygiene, and public health that would be familiar to any current student of global health. Initially, this specialization had emerged within colonial governments, but quickly it split off into an independent academic profession. In part, this reflected stronger career incentives and opportunities for greater prestige [42, 48, 49]. The insights gained from scientific study were codified and advanced through elite, internationally oriented academic networks that functioned along lines of shared experiences and expertise in colonial settings, which were largely separate from existing domestic medical networks. Both Ross and Laveran were awarded Nobel Prizes for their malaria work (1902 and 1907, respectively), and Ross in particular spent much of his subsequent career complaining that physicians doing tropical medical research were not recognized or remunerated properly for their leading role in the colonial enterprise [42]. In launching the *Société de Pathologie Exotique* in Paris in 1908, Laveran cited facilitating colonial expansion and protecting the metropole as primary motivations [50], much as Manson had done a few years before at the foundation of the London School of Tropical Medicine [51].

In metropolitan consciousness scientific progress and service to empire blended easily with notions of humanitarianism, saviourism, charity and other paternalistic concepts that helped justify colonialism and disguise its non-merit hierarchies [52]. As Nancy Rose Hunt examines in the case of Belgian occupied Congo, metropolitan groups enhanced their own status by organizing around perceived health deficiencies of Indigenous people. These efforts fit colonial narratives of metropolitan superiority and charity as they sought to enhance the Indigenous labor force available for exploitation [53]. This pattern of mainly self-interested intervention by elite metropolitan researchers and clinicians has been noted in the global health era, as well [54].

Although these and similar schools were all in rapidly modernizing cities uplifted in a golden age of public health institution building and infrastructure construction [55], tropical medicine did not emphasize that knowledge or prominently feature its fundamental advances: sanitary sewerage, water filtration, or public administration [24, 28]. Instead, tropical medicine formed around laboratory and clinical aspects, particularly bacteriology, parasitology, and entomology. As Deborah Neill has analyzed, topical medicine was constituted through well-connected, elite, cosmopolitan networks that were cohesive across many European settings. They constituted their authority through international mutual reinforcement and did not connect closely with domestically oriented colleagues in their own countries [24]. Tropical medicine was one of the early “epistemic communities” that emerged in the late 19^th^ century around international research and policy concerns [56].

(The story of tropical medicine in the United States is roughly similar, with the founding of the American Society of Tropical Medicine in 1902 and the first independent school (Tulane) in 1912, although yellow fever was much more prominent due to American imperial ambitions in the Caribbean, and in Central and South America [57–60]).

### A basis for racist inequality

Scientific findings on the details of malaria transmission quickly percolated into colonial policy. The close connection between academic tropical medicine and colonial administration came from shared experience, common goals, and mutual dependence. Many or all members of the emerging profession had been in colonial service or colonized places, wanted to advance colonialism, and wanted to solve the same problems in the same places as their colonial service counterparts. Hence, within a few months of retiring from service in India and joining the Liverpool School, Ross headed to West Africa on a malaria expedition at the request of the Colonial Office [61]. Ross and his colleagues recommended abandoning older, more expensive drainage strategies in favor of a more targeted attack on only Anopheline mosquitoes and their habitat, which would be cheaper, easier, and have the same effect on malaria as more general measures, they argued [61]. The recommended strategies also required the expertise of Ross and his colleagues, whereas more environmental measures did not. New knowledge about malaria transmission by Anophelines and the racist perception of Africans as a reservoir of disease led to an official Colonial Office policy of segregated living as of 1901. The construction of hill stations, separated European-only neighborhoods in cities, and separated lodging areas on plantations were quickly pursued throughout the British Empire [62, 63].

Racist segregation policies quickly led to a divergence of recommended malaria control measures, depending on whose well-being was perceived to be at stake. Much emphasis was placed on the appropriate location and construction quality of homes for Europeans. For example, in Freetown, official plans called for housing for Europeans at high altitude, at least a half mile from any Indigenous person’s dwelling, and featuring extra-large windows to admit salubrious breezes. This led to a conflation of segregation and safety—safety defined with sole reference to European concerns—while deploying the benefits of malaria knowledge almost exclusively for Europeans [63]. Racism underpinned the views of many in tropical medicine, such as Liverpool expedition member John Todd, whose letter home is quoted by Neill, “the climate is glorious and some day it will be crowded with white-skinned people who will wonder why their forefathers thought Africa so unhealthy” [24]. Similarly, colonial governments routinely dispensed quinine tablets for Europeans, but did not do so for Indigenous people. Writing in South Africa, one colonial medical officer explained the conditions under which malaria control efforts would be extended to Native people:

The unscreened native hut is…a very great danger to the farmer, particularly when it is only a matter of a few hundred yards or less away from his home. These squatter native families on Transvaal farms are a malarial menace on account of their being the reservoir of infection for the newly-born mosquito vector seeking its first blood-meal. Generally, we advise farmers to keep such huts a good distance—at least a mile away from European homes. Where this is impracticable, these native huts should at least be sprayed daily [64].

The narratives of colonial malaria control did not give prominent attention to the ways in which colonialism itself was responsible for increasing the distribution and worsening the consequences of the disease. As historian Randall Packard has investigated, large-scale agricultural practices, labor conscription, and forced migration all had disastrous consequences for the prevalence of malaria. Irrigation and dam projects created vector breeding sites where none had existed, forced migration spread the parasite by mixing infected and naïve populations, poor nutrition and poverty increased susceptibility, and land seizures forced Africans to live in unhealthy geographies they had previously avoided [26]. To a large extent, modernizing agriculture had produced similarly disastrous increases in malaria wherever it had existed, including in Southern England in the early 1800s. But the burden fell over the following decades with generalized improvements in environment and living standards, hygiene, and nutrition—all before the scientific insights of Laveran, Ross, and Manson [65]. The same thing had happened in the Southern United States, as well [66]. Following Packard’s analysis, these developments happened organically under normal conditions, but under colonialism, the continual extraction of economic surpluses, the enforcement of trade terms that disadvantaged Indigenous people, and governments that prioritized European interests all enforced an ongoing state of under-development that both promoted malaria and precluded public health responses [26].

## Conclusions: Colonial continuity in the global health era

In the global health literature of today, narratives typically cast malaria as a historic scourge described in ancient texts from China, Egypt, and by Hippocrates himself in Greek antiquity [67]. Clinical and technical descriptions of malaria are also common, characterizing the disease as the result of infection by a protozoa, *plasmodium* [68]. Others have favored climactic explanations to depict malaria uncritically as a feature of some natural environments, part of a pattern that allows global health elites to overlook legacies of extraction [69]. The impression created by such narratives is that malaria has always existed and always expressed a terrible toll wherever it has been found. This perspective hides a more complete truth. Malaria is indeed an historic disease, but the malaria of old was vanquished by ordinary and organic processes that characterize development. Meanwhile, the current malaria discourse mainly focuses on the parasite and the vector, and consistently excludes a focus on environmental measures. This is reminiscent of the solutions proposed by Ross and colleagues in the 19^th^ century, which appear to be self-serving as the evidence that environmental strategies worked well for malaria control is largely ignored. The pattern of malaria as we see it today, on the other hand, were produced by colonialism, and the study of malaria as we know it now was intended to protect colonial interests, not to help Indigenous people or defeat the disease more broadly. Hence, the academic study of malaria played a crucial role in sustaining the spread of disease, providing ways for Europeans to colonize more effectively, even as their activities fostered a new large-scale ecology of unending malaria for Indigenous people.

As pioneered by Ross, Laveran, and many others, the scientific study of malaria yielded greater pay and prestige as part of an international academic enterprise, as opposed to within government or even colonial service. This established London, Liverpool, Paris, Antwerp, Berlin, Boston, Baltimore, New Orleans, and other metropoles as the enduring centers of malaria research even though the disease was already defeated in those places, or soon would be. This shows again how knowledge about malaria, as constituted under colonial patronage, was designed more to win prestige for researchers and to selectively manage the disease’s threat without actually investing in the development that had rendered it unimportant in the cities where these same schools were founded. Thus began the long tradition of metropolitan researchers making frequent travel to malarious areas for reasons that were based on self-interest. These origins show why the centers of malaria research have never moved toward the areas where malaria is a problem, and they explain why local knowledge about malaria and affected communities is not valued in the research process. Colonialism was prosecuted over the objections of Indigenous people, and so too was its research.

Data is the lifeblood of academic work on malaria, and control of its collection, ownership, and analysis is closely guarded by Northern institutions, even though most of it must be gathered in endemic areas of Africa. In this light, the commonly known donor and international agency preoccupation with data and measurement can be viewed as an effort to maintain control over a primary resource for Northern schools and departments engaged in global health. This practice is in some tension with the publicly-stated objective of improving the quality of decision making because it influences who is involved in that process and what can be decided. The first consequence is to maintain the prominence of outside specialists who have no formal legitimacy in state-citizen deliberations but wield significant influence due to their financial and epistemic resources. The second is to focus attention on the disease, distracting from the environmental management and general development strategies that donors have used for themselves.

The long history of systematic data collection by donors has been one means of maintaining the primacy of Northern institutions in debates about the global South, for instance, the US Government has been funding data collection efforts since at least the 1950s [70], including the World Fertility Survey starting in the 1970s [71] and the Demographic and Health Surveys since 1984 [72]. It is important to ask who designs the methods, who shapes the questions, who controls the data, and who benefits as a result. In the colonial period, Indigenous people were sometimes the subject of study and sometimes participated in collection, such as by catching insects for entomological surveillance, much as they now enumerate surveys and facilitate the legwork of research in languages and cultures that metropolitan specialists never bothered to learn. But however integral they were to the research process, Indigenous people were almost never permitted to participate in the analysis and decision making that followed. It would appear that these patterns are little changed; a recent study found that about 53% of global health papers about Africa have a first author from the country of interest, but this proportion fell to only 23% for papers that had any author from the US, UK, or Europe [73]. In collaborations between Africans and people based in the colonizing countries, it is the colonizers who end up with the lion’s share of the credit, even though they have no lions of their own, counting only those previously stolen from the sub-Sahara now interned in zoos.

Aside from academic interests, the primary goal of colonial malaria control was to facilitate economic extraction. It is troubling that the dominant strategies in malaria control today are privately produced products manufactured elsewhere: bednets and pharmaceuticals. Swamp drainage, housing improvement, and other generalized aspects of public health and development defeated malaria in rich countries, but are not common aspects of donor programs now. Even residual insecticide spraying, a long-proven technique, is no longer in favor. Dominant interventions are thus those that involve a commodity purchase and focus on areas where foreign academics would have an advantage—pathogens and other technical factors less reliant on local knowledge, as would be the case with development questions, behavioral patterns, politics, or the social contract. We must ask if these patterns reflect the continuing dominance of private sector interests and elite academic career incentives over the health of people at risk of malaria.

Rethinking malaria today thus requires grappling with the colonial shaping of malaria and malaria control. Colonialism is so central to the creation of both malaria and its related academic enterprise that it is impossible to decolonize without rethinking every underlying principle and relationship. Although few or none in global health today would identify in support of the colonial project, the roads on which we all walk were built for extractive purposes and still embody unquestioned inequalities of power and privilege. The concepts and institutions of colonial malaria are embedded deeply in the current efforts related to this and other problems. Indeed, many people do not recognize the reach of the colonial roots, which makes efforts at decolonizing that much more challenging.

To consider decolonization means to ask questions about the world in which we live and its dominant patterns, and to consider alternative concepts for thinking about malaria and malaria control. Positions of privilege can be useful for raising such questions, but the identification of solutions requires moving the locus of discussion from the metropoles to affected countries. To rethink malaria in this context requires changing who is most central in the discussion and altering the lines of accountability, and creating new concepts of disease and control; It requires reversing the direction of control, from funders and other agencies in the global north to those in the endemic south, and engaging the people whose lives are endangered by malaria. Fundamentally, decolonization means rethinking and restructuring the governance relationships that shape decisions about malaria. Decolonization is not fundamentally a rejection of knowledge accumulated under colonial arrangements, nor a return to pre-colonial conditions; instead it is a question of how we change objectives and accountabilities in favor of development and autonomy, and how we use that knowledge to move away from the production of inequality and dependency.
